# Ten tips for the management of vascular access in hemodialysis patients

**DOI:** 10.1093/ckj/sfag366

**Published:** 2026-10-10

**Authors:** Uberto Percudani, Marco Delsante, Giordano Pastori, Meilad Shakkour, Enrico Fiaccadori, Andrea Bandera

**Affiliations:** Nephrology Unit, Department of Medicine and Surgery, University Hospital of Parma, Parma, Italy; Nephrology Unit, Department of Medicine and Surgery, University Hospital of Parma, Parma, Italy; Nephrology and Dialysis Unit, Hospital of Belluno, Belluno, Italy; Nephrology Unit, University Hospital of Verona, Verona, Italy; Nephrology Unit, Department of Medicine and Surgery, University Hospital of Parma, Parma, Italy; Nephrology and Dialysis Unit, Hospital of Belluno, Belluno, Italy

**Keywords:** arteriovenous fistula, arteriovenous graft, chronic hemodialysis, dialysis, vascular access

## Abstract

Vascular access management represents one of the central challenges in hemodialysis care, requiring the nephrologist to navigate a complex and dynamic process that begins well before the first dialysis session and continues throughout the patient’s entire treatment trajectory. This challenge is further amplified by the growing age and comorbidity burden of the hemodialysis population, and by the recent shift toward high-convection-volume hemodiafiltration, which depends on a reliably functioning access. Drawing on current evidence and clinical experience, this narrative review argues that optimal outcomes depend not on any single intervention, but on a coherent, longitudinal approach integrating timely planning, careful patient selection, surgical precision, and structured follow-up. In this context, the nephrologist plays a pivotal coordinating role, and assuming ownership of this field through continuous training, multidisciplinary engagement, and education of patients and colleagues remains essential to improving outcomes along the entire course of kidney disease.

## INTRODUCTION

The number of prevalent hemodialysis patients has increased by 104% over the past 30 years [1], and in countries such as the United Kingdom, a 75% increase in the hemodialysis population is projected by 2032 [2]. The mean age of prevalent patients has also increased by 44%, particularly in high-income macro-regions [1]. In Europe, 83% of patients referred for kidney replacement therapy start on hemodialysis, particularly those aged >75 years [3].

A functioning vascular access represents the cornerstone for delivering adequate hemodialysis treatment, but age, frailty, and comorbidities make the proper management of vascular access more challenging [4], although a native arteriovenous fistula (AVF) particularly if proximal or an arteriovenous graft (AVG) remains, in some cases [5], the best option even in this patient population [4, 6, 7].

For the nephrologist involved in hemodialysis care, it comes as no surprise that vascular access represents one of the main challenges in patient management, particularly now that the recent shift toward increased use of hemodiafiltration, following the CONVINCE trial [8] and subsequent meta-analyses [9], requires a well-functioning access capable of achieving the convection volume target of 23 l per session, beyond which a survival benefit is observed.

It is from these considerations that, in our view, the rationale for this review stems, the aim of which is to bring this topic back to the attention of the nephrology community and to provide a starting point for further exploration of the subject. (Fig. 1)

**Figure 1:**
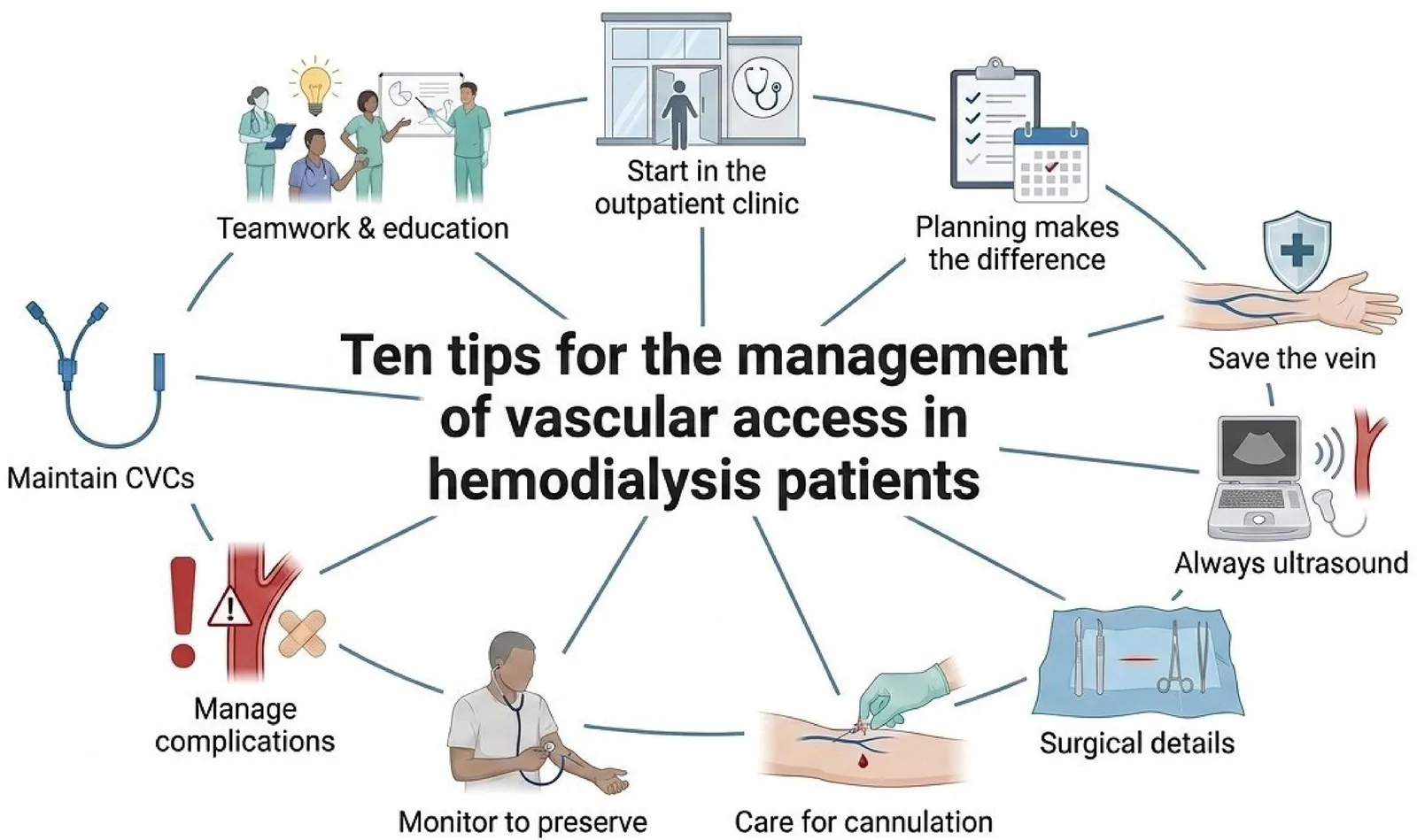
Ten tips for the management of vascular access in hemodialysis patients.

## TIP 1: START IN THE OUTPATIENT CLINIC

Before planning vascular access creation, consideration should be given to whether the patient might instead be a candidate for pre-emptive transplantation [10], for peritoneal dialysis [11] particularly when the nephrologist is responsible for peritoneal catheter placement [12] or when logistical concerns regarding feasible hemodialysis are present [13], or for conservative management. Conservative management may be the best alternative in older and comorbid patients [14–16].

There is no consensus regarding the ideal timing for access creation. The 2024 KDIGO guidelines suggest, as a practice point, that patients with a 2-year risk of kidney failure >40% (e.g. as calculated using the Kidney Failure Risk Equation [17, 18]) or an estimated glomerular filtration rate (eGFR) <15 ml/min should be referred for vascular access planning [19]. In an observational study on a large Swedish cohort, Lundström *et al*. [20] argue that adopting the 2-year risk of progression to dialysis may be a superior approach for correct timing, as confirmed by data from other cohorts [21]. However, other guidelines suggest different cutoffs, based on eGFR [22, 23] or on early referral 12 months before hemodialysis initiation [24] (Table 1).

**Table 1:** Different guidelines for vascular access referral.

	JSDT 2011	GEMAV 2017	UKKA 2023	KDIGO 2024
Timing of referral for vascular access planning	VA construction should be considered when eGFR is less than 15 ml/min/1.73 m^2^ (CKD stages 4 and 5) as well as taking into account clinical conditions (O). In patients with diabetic nephropathy, who have a tendency to show overhydration, VA construction should be considered at a higher eGFR (O).	We recommend that the creation of vascular access be considered in patients with progressive chronic kidney disease when eGFR is less than 15 ml/min/1.73 m^2^ and/or when estimating that dialysis will be needed in 6 months	Whilst optimal vascular access timing depends on patient and institutional factors, we suggest access referral, and if suitable fistula formation, are appropriate for any adult or child planning hemodialysis and likely to start within 12 months,	Consider planning for preemptive kidney transplantation and/or dialysis access in adults when the GFR is <15–20 ml/min per 1.73 m^2^ or risk of KRT is >40% over 2 years.

JSDT, Japanese Society for Dialysis Therapy; GEMAV, Spanish Multidisciplinary Group on Vascular Access; UKKA, The UK Kidney Association; KDIGO, Kidney Disease Improving Global Outcomes.

Several studies have shown that adequate predialysis follow-up significantly reduces the likelihood of being started on dialysis with a temporary or permanent central venous catheter (CVC), thereby reducing mortality and morbidity [25–28]. In a recent retrospective study on a large cohort of 146 967 patients, initiating hemodialysis with a native or prosthetic AVF was associated with a 25% lower risk of mortality, although this finding is likely influenced not only by the type of access chosen but also by patients' baseline health status [29–31].

These observations point to the central role of the nephrologist as the leader of the multidisciplinary vascular access team: it is indeed the nephrologist who is in contact with the patient when the dialysis modality is chosen, refers the patient for access creation at the optimal time, and who will manage the access once it has been created.

## TIP 2: PLANNING MAKES THE DIFFERENCE

Since the 2019 KDOQI guidelines, vascular access has been conceptualized in terms of a life-PLAN, shifting the focus beyond a Fistula First approach toward a shared decision-making process summarised by the principle: ‘the right access, in the right patient, at the right time, for the right reasons’ [32, 33]. A dynamic strategy is proposed encompassing both short- and long-term planning, beginning with an assessment of whether the patient is a suitable candidate for a native AVF, ideally on the nondominant side, and, if not, whether a prosthetic AVF is feasible or whether it could serve as a bridge to a native access; otherwise, taking patient preferences into account, a permanent CVC remains a viable option [34]. Table 2 summarizes, in patient-friendly language, the characteristics of the different vascular access types that may be discussed with the patient at the time of access selection. Our preference is to pursue a native access even when technically more demanding, such as a brachiobasilic AVF with basilic vein superficialization, before considering any prosthetic option, including in elderly patients, as suggested by the Spanish guidelines [22].

**Table 2:** Shared decision-making visual aid.

Access type	Durability	Infection risk	Patient perception	Daily activities
AVF	Depends on several variables, such as anastomotic site, patient age, sex, or race; generally the most durable access. May require reintervention to assist maturation and, subsequently, to maintain function. Usually allows for better toxin clearance.	Low infection risk, increased in case of buttonhole cannulation	Generally allows for a shorter time to commence the dialysis session. Cannulation is associated with pain, particularly at the outset of treatment, the extent of which depends on cannulation technique and may be mitigated by pharmacological and nonpharmacological interventions. Postoperative numbness may occur. Aneurysmal evolution may alter arm appearance over time, although this can facilitate cannulation.	Requires regular thrill monitoring. Some limitations apply to daily activities, including avoiding sleeping on the fistula arm, wearing tight sleeves that may act as a tourniquet, carrying heavy weights, and engaging in contact sports that could traumatize the AVF; blood pressure measurements, blood sampling, and intravenous injections should also be avoided on the AVF arm. Overall, allows for a more active lifestyle.
AVG	Intermediate durability; requires reintervention to maintain function more frequently than AVF, but is generally associated with lower initial failure rates and catheter dependence	Intermediate infection risk; systemic infection may occur and can require graft removal.	Same as AVF above	Same as AVF above
CVC	Least durable; may require replacement due to complications or malfunction. Predisposes to central vein stenosis, potentially limiting future vascular access options.	Highest infection risk, with possible localization to the heart, bone, or brain.	Depending on catheter position, may be easier to conceal than AVF/AVG.	Requires attention to keeping the central line covered and secure. Extra care is needed while showering, swimming and bathing should be avoided, extra care when interacting with children.

AVF, arteriovenous fistula; AVG, arteriovenous graft; CVC, central venous catheter.

At the time of planning, a thorough clinical assessment is essential. This includes a detailed medical history including female sex [35], age, and relevant comorbidities such as diabetes, underlying nephropathy, oncological history, peripheral arterial occlusive disease, or heart failure; prior vascular access procedures, previous CVC, peripherally inserted central catheter (PICC), midline catheter, or pacemaker placement; known coagulation disorders; and a complete medication history with particular attention to anticoagulant or antiplatelet therapy [36], which may need to be reviewed as part of preoperative planning. Physical examination should ideally be performed in a room at adequate temperature to minimize vasoconstriction and allow accurate assessment of superficial vessels. Assessment of the arterial compartment includes evaluation of peripheral pulses, bilateral upper limb blood pressure measurement, and the Allen test (Fig. 2). The venous compartment is assessed after tourniquet application, identifying visible veins, ideally superficial with a relatively linear course, with percussion of the vessel allowing evaluation of transmission and therefore patency [22]. The optimal approach incorporates upper limb color Doppler ultrasound assessment, which will be discussed in detail in tip 4 of this review.

**Figure 2:**
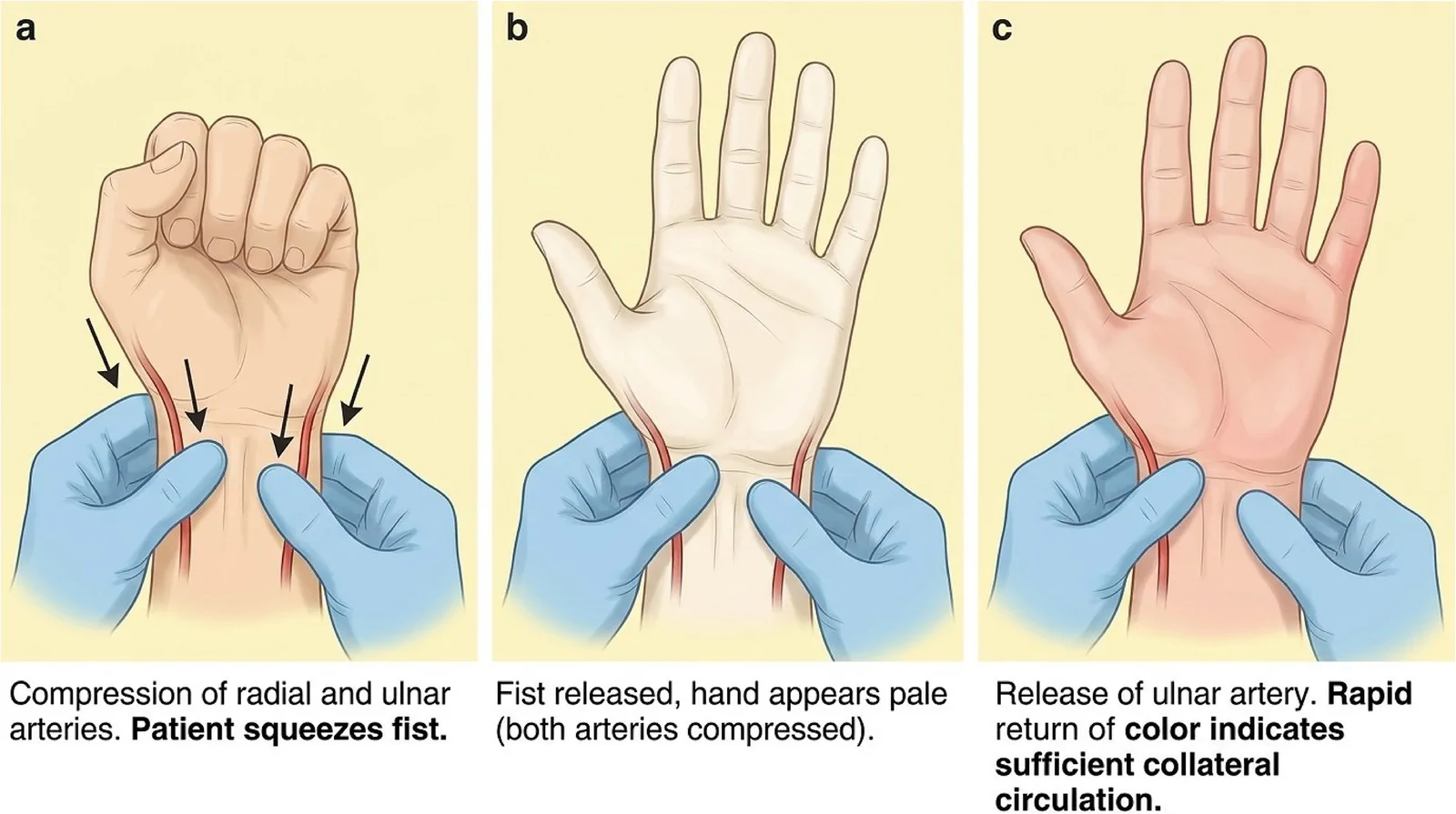
Allen test to assess patency of palmar arch and collateral circulation of the hand.

Ideally, when the patient is discharged following the preoperative assessment, the surgeon should have formulated both a plan A and at least a plan B for the surgical approach. The goal is to create an access that functions with the fewest reinterventions possible, avoiding primary failure, the incidence of which reaches 24.7% according to a recent meta-analysis [37], rather than always pursuing the most distal access [38–40].

## TIP 3: SAVE THE VEIN

We have chosen to address this point separately in order to highlight its importance: it is a detail that makes a real difference, particularly when patients are educated and actively engaged in this simple precaution, in the spirit of campaigns such as Save the Vein in the UK [41].

In patients at risk of end-stage kidney disease, preserving venous capital is important, particularly once the preferred site for vascular access creation has been identified [42]. In these patients, venepuncture and peripheral venous catheter insertion proximal to the wrist should therefore be avoided, with specific attention to preventing the use of PICCs [43, 44]. Regarding CVCs, subclavian access is associated with the highest risk of central venous stenosis [45]. In patients who are likely to be listed for kidney transplantation in the future, such as younger individuals, femoral CVC insertion should likewise be avoided.

## TIP 4: ALWAYS ULTRASOUND

Color Doppler ultrasound enables a rapid, cost-effective, and noninvasive assessment of the vascular anatomy of the upper limbs, both at the time of feasibility evaluation and access selection [46, 47] and immediately before skin incision to confirm the chosen site. It is not uncommon for pre-incision assessment to lead to a change in surgical strategy, sometimes because a previously favorable anatomy is no longer present, or because a new viable option has been identified.

The examination should be performed by the same operator who will carry out the procedure, and should incorporate relevant clinical information: for instance, how long before the initiation of renal replacement therapy the assessment is being made, or whether the patient is being evaluated on a dialysis day, as in the postdialysis setting significant ultrafiltration may result in a less favorable appearance of the venous capital.

Assessment begins with the arterial compartment: the brachial artery, the level of its bifurcation, and the peak systolic velocity sampled at the radial artery. Indeed, small-caliber arteries with a peak systolic velocity of 25–30 cm/s may still represent a good inflow option [48]. This is followed by venous assessment, performed with the aid of a tourniquet to evaluate venous distensibility, starting from the cephalic vein of the forearm, the median vein, assessing the presence or absence of a perforating vein at the antecubital fossa, the basilic vein, and the cephalic vein of the upper arm, tracing the course as far as the subclavian vein where possible. Ideally, one seeks a vein with a straight course, compressible and therefore free of thrombosis with adequate outflow. Vessel diameter should be measured in transverse section, applying the least possible pressure. Conventionally, a minimum arterial diameter of 2 mm and a venous diameter of at least 2–2.5 mm for AVF, or 4 mm for AVG, are sought. Assessment of the distance between vein and artery, and where relevant, the depth of the vein are also useful in determining feasibility [49].

We therefore consider the acquisition of ultrasound skills to be the essential starting point for any operator approaching the field of vascular access creation for hemodialysis.

## TIP 5: SURGICAL DETAILS

AVF creation is a procedure in which small details make the difference [50], a demanding operation that should not be left to inexperienced surgeons [51, 52]. In this context, several subtle technical variations may lead to equally satisfactory outcomes, with meticulous attention to detail as the common denominator [53].

Our technique shares several similarities with that described by Konner in several of his publications. The skin incision should be kept small and oriented longitudinally, so as to avoid injury to lymphatic vessels and to favor wound healing [54]. Dissection proceeds in layers, with the aid of monopolar diathermy to maintain a clean operative field, deepening the exposure progressively. Careful preoperative ultrasound assessment will have identified the location of both vein and artery before the incision is made. The angle between artery and vein should be acute [55], but above all the choice of anastomotic site and configuration, end-to-side or side-to-side, must be dictated by the anatomy as encountered intraoperatively; it is essential not to force vessels into unnatural positions, to reduce the risk of torsion. Regardless of the anastomotic technique employed, the artery should be manipulated as little as possible, and the vein mobilized only over the length strictly necessary, with minimal removal of adventitia [54].

Before completing the anastomosis, the thrill should be assessed; if unsatisfactory, it may be helpful to introduce an olive tipped needle or vascular dilator into the artery to relieve vasospasm, and if needed into the vein to remove any valves, which are particularly likely to be present in the perforating vein. Generally, a low-flow AVF suggests an inflow problem, whereas a pulsatile AVF, indicating an attempt to overcome resistance, points to an outflow problem, such as an unrecognized stenosis. It is therefore important to inspect the course of the vein, eliminating any external compression, and to ensure a smooth angulation where the artery meets the vein.

## TIP 6: CARE FOR CANNULATION

The success of an AVF is ultimately measured by its suitability for cannulation and effective use. The decision regarding AVF maturity has conventionally been based on the rule of 6s: assessment at least 6 weeks after creation, outflow vein diameter of at least 6 mm, depth from the skin surface of less than 6 mm, and blood flow of at least 600 ml/min. However, to reduce the number of unnecessary interventions on AVFs that are in fact mature, the University of Alabama at Birmingham (UAB) criteria have been proposed, according to which AVFs with a smaller vein diameter (down to 4 mm) and lower blood flow (≥500 ml/min) may also be considered mature [56, 57]. A pragmatic clinical approach would rather be to consider an AVF mature when it can be consistently and adequately cannulated with two needles [58].

Cannulation should be performed by dedicated and trained nursing staff [59], and it is essential that it be preceded by a physical examination of the access [60]. An extension of this principle is that operators responsible for cannulation should develop skills in ultrasound-guided cannulation, including by practicing on straightforward AVFs to be prepared when faced with more challenging ones [61, 62]. The optimal cannulation technique is the rope-ladder approach, which exploits the full length of the vein; in selected patients with a native AVF, buttonhole cannulation may be considered [63, 64], while area cannulation, associated with the highest rate of complications, should be avoided when possible [65].

At the time of cannulation, it is important to remember that the blood flow achievable during dialysis varies according to needle gauge. The recommendation is to begin cannulating the fistula with smaller-gauge needles and, once adequate maturation has been confirmed, to progress to larger gauges.

Several practical precautions may appear trivial but are effective in reducing complications such as infiltration: the needle should be secured to the patient’s arm rather than to the bed; needles should be inserted with the bevel facing upward; and both needles should be oriented in the direction of flow. The only indication for retrograde cannulation is when the cannulation segment is short; in any case, needles should never be placed at or near the anastomosis. A range of strategies are available to mitigate cannulation pain and anticipatory anxiety, including the application of topical anesthetic agents such as lidocaine, with or without prilocaine (e.g. EMLA cream), although strong evidence is lacking; a recent meta-analysis suggests that some non-pharmacological measures, such as cryotherapy combined with visual distraction, visual distraction alone, or aromatherapy, may be superior to pharmacological strategies [66]. It is equally important, in cases of difficult cannulation, to refer to an experienced operator.

To facilitate the work of nursing staff, it may be helpful to create an access map with guidance on cannulation sites and their rotation.

## TIP 7: MONITOR TO PRESERVE

The first participant involved in access monitoring is the patient; educating patients to recognize potential problems with their AVF is therefore the essential first step. Table 3 summarizes the key warning signs that patients should be able to identify. Examples of this approach include educational materials developed by Dorough and colleagues, consisting of an informational booklet and a video created in direct collaboration with patients themselves [67, 68]. It is essential that patients be informed to avoid tight sleeves, watches, or bracelets that could occlude the AVF; similarly, they should avoid contact or traumatic sports, while continuing to use the AVF arm for normal daily activities and other sports. Patients should also avoid sleeping on the AVF arm and should not undergo blood pressure measurements, blood sampling, or injections in the AVF arm. The access arm should be washed with soap and water prior to cannulation; after needle removal, manual compression should typically be applied for 10–12 min, avoiding excessively compressive dressings, and once home, dressings can be safely removed after 3–4 h.

**Table 3:** Warning signs that warrant contacting the dialysis centre.

Warning sign	Clinical significance
Redness, pain, discharge, swelling, or fever	May indicate access infection
Rapid change in the skin overlying the fistula e.g. thinning or discoloration of the skin (e.g. dark/black areas) over an aneurysmal segment	May indicate that an aneurysmal segment is at risk of rupture
Absent thrill, or thrill that has decreased in intensity	May indicate access dysfunction or impending thrombosis
Prolonged bleeding through the dressing after the dialysis session	Requires immediate re-compression for at least 5–10 min, followed by application of a new dressing; if bleeding does not stop, urgent presentation to the emergency department may be necessary
Altered sensation, coldness, or pain in the AVF hand or limb	May indicate access-induced ischemia

The second key contributor is the nurse, who, before using the access, must systematically enquire about any problems the patient may have experienced during the interdialytic period, and must be able to perform a structured physical examination based on the look-listen-feel approach. The nurse also reports information derived from the dialysis monitor such as pressures, alarms, and recirculation as well as any inability to complete the session and prolonged bleeding time following needle removal.

The physician completes the monitoring process with a clinical and laboratory assessment of the patient, for instance in response to signs suggestive of underdialysis [42].

When abnormalities are identified within this monitoring framework (Fig. 3), ultrasound evaluation of the access may be indicated to exclude complications and, where appropriate, to initiate surveillance of specific lesions such as stenosis [69]. This reassessment also provides an opportunity to plan a new access for the patient if required.

**Figure 3:**
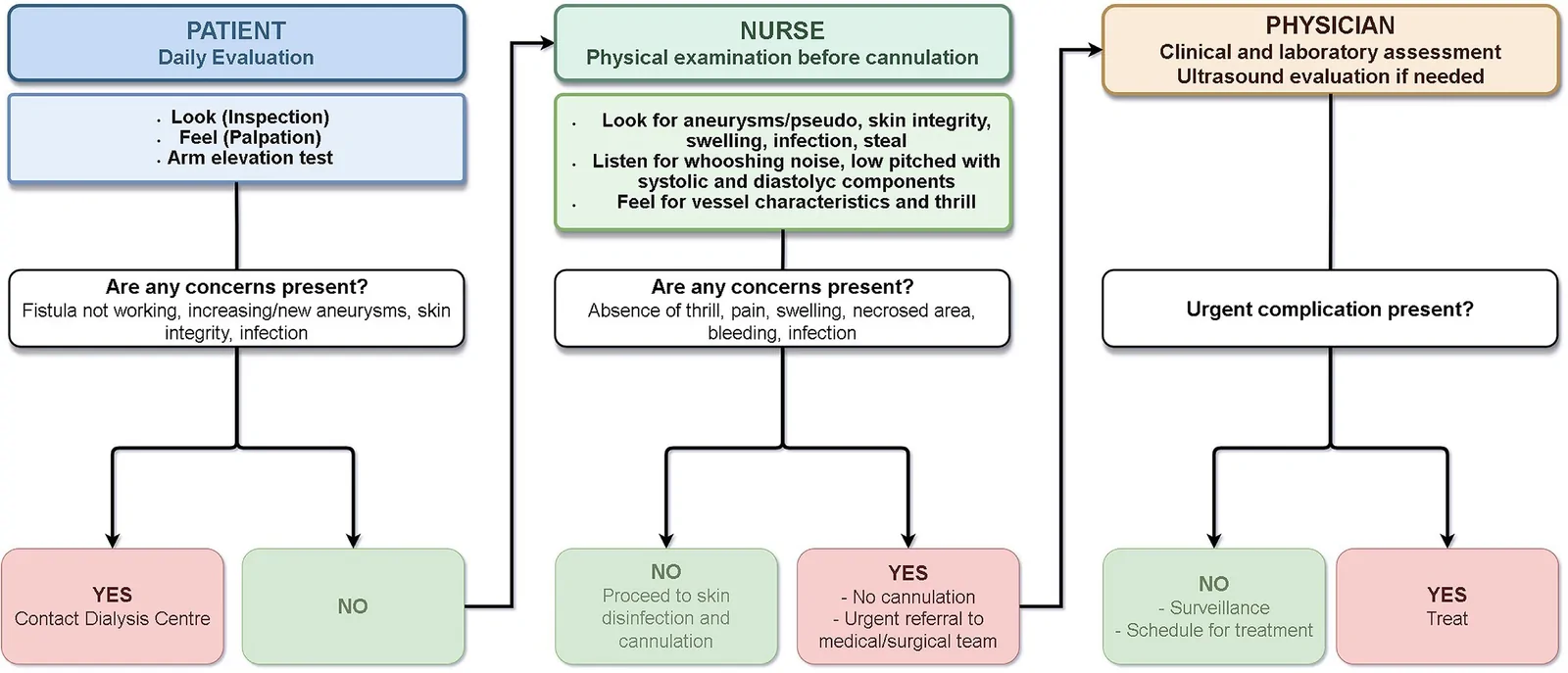
Monitoring framework.

## TIP 8: MANAGE COMPLICATIONS

When a complication arises [70], the first question to address is whether it requires urgent intervention or whether a period of monitoring prior to treatment is feasible. Urgent conditions include: necrotic lesion overlying an aneurysmal or pseudoaneurysmal segment; arterial pseudoaneurysm resulting from inadvertent arterial puncture; thrombosis; ischemic monomelic neuropathy; and infection of a prosthetic AVF.

Management of a necrotic lesion requires excision of an ellipse of overlying skin, with concomitant aneurysm reduction in the case of a native AVF or repair of the defect in the case of a prosthetic AVF [71]. Management of an arterial pseudoaneurysm requires proximal and distal control of the artery, evacuation of the aneurysmal sac, and subsequent arterial repair. Thrombosis of an AVF or AVG should generally be addressed within the first 24–48 h, and requires mechanical thrombectomy using a Fogarty catheter, followed in most cases by endovascular treatment of the downstream stenosis, which in the majority of cases represents the underlying cause of dysfunction [71]. Management of severe ischemia requires ligation of the AVF, while infection of prosthetic AVF necessitates graft removal.

Regarding the management of non-urgent complications, we consider, although controversial, surgical correction of venous stenosis to be the first-line approach. Several techniques are available: when the stenosis is short and the vein of sufficient length, resection of the stenotic segment with end-to-end anastomosis between the two venous stumps is feasible; alternatively, patch venoplasty may be performed by incising the stenotic wall and applying a patch harvested from an adjacent native collateral vein [72], or using a Dacron patch of the type employed in carotid endarterectomy [73, 74]. We reserve endovascular treatment, percutaneous transluminal angioplasty (PTA) with or without stenting, as a first-line approach for stenoses that are not readily amenable to surgical correction, such as graft-vein anastomotic stenoses in prosthetic AVFs or central venous stenoses.

The management of high-flow AVF and hemodialysis access-induced distal ischemia (HAIDI) presents several layers of complexity; a detailed discussion of the respective indications and surgical techniques lies beyond the scope of this review, but it has recently been very well addressed in the review by Coscas *et al*., summarized in Fig. 4. Worth recalling in this context is a clinical entity that is sometimes overlooked, venous HAIDI caused by venous hypertension and congestion at the hand level [75], whose management requires promotion of venous return for instance by creating a veno-venous anastomosis with a different superficial vein to augment outflow.

**Figure 4:**
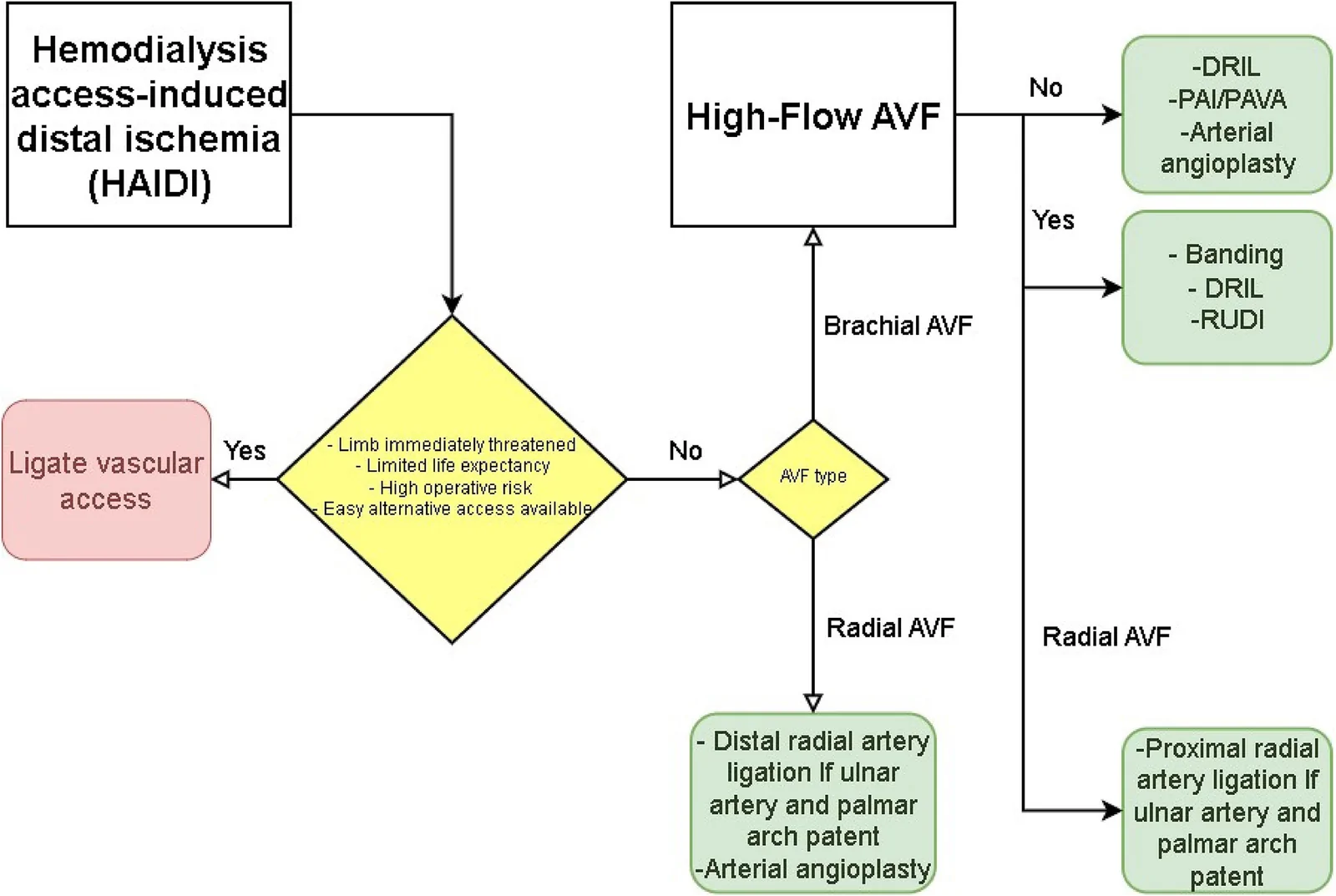
Managment of high flow and hemodialysis access-induced distal ischemia based on "Coscas R, Jayet J, Basceken V, Javerliat I, Coggia M. Prise en charge des complications évolutives des abords vasculaires d’hémodialyse. EMC—Techniques chirurgicales—Chirurgie vasculaire 2024;40(3):1–19 [Article 43–029-S]. DRIL, distal revascularization and interval ligation; RUDI, revision using distal inflow; PAI, proximalization of arterial inflow; PAVA, proximal arteriovenous anastomosis.

## TIP 9: MAINTAIN CVCs

When managing a patient with a CVC, it should be kept in mind that they are generally at higher risk of complications. The most obvious measure to reduce CVC-related complications, infections being the foremost concern, would be to minimize CVC placement whenever possible. However, in some patients a CVC remains the preferred access, and in these cases many of the considerations already discussed continue to apply. Frequent training of both the patient and the nursing staff who handle the CVC is essential [76], in the same way as training is provided for peritoneal catheter management. Although the evidence supporting quality improvement interventions remains limited, the literature suggests that even simple adherence to a rigorous ‘scrub the hub’ protocol by nursing staff is associated with a reduction in the incidence of catheter-related infections [77].

The preferred insertion of a permanent CVC in the right internal jugular vein, with the tip positioned in the upper portion of the right atrium, is driven by anatomical considerations that not only allow higher blood flow rates to be achieved [78], but also reduce the risk of central venous stenosis, which typically occurs at sites where the catheter contacts the vessel wall. In contrast, following the tortuous course of the left jugular vein increases the likelihood of catheter-induced stenotic lesions. In our experience, softer catheters, such as those made of silicone, may represent an adequate solution.

In clinical practice, when inadequate blood flow during a dialysis session is encountered, initial management includes flushing the catheter with normal saline, repositioning the patient [79], and, if needed, reversing the blood lines [24]. These measures, however, rarely provide a durable solution, and thrombolytic agents are frequently required [80]. If blood flow remains insufficient despite these interventions, CT angiography or conventional angiography of the central vessels should be requested to detect the presence of a fibrin sheath or central venous stenosis, and appropriate treatment should be undertaken.

## TIP 10: TEAMWORK, EDUCATION AND THE PATIENT AS A PARTNER

Vascular access management relies on collaboration among different figures within a dedicated vascular access team, including the dialysis nurse, the nephrologist, the interventional radiologist, and the vascular surgeon [81–83], although at present there is insufficient evidence that a multidisciplinary approach can increase AVF utilization at the expense of CVC use [84].

Given that several points, particularly those of an organizational nature, collected in the most recent guidelines on vascular access management are based on expert opinion, ongoing dialogue among stakeholders remains valuable. This includes sharing perspectives, engaging with the most recent literature, and actively participating in the broader discussion.

To foster a culture centered on vascular access management, it is essential to continue training future generations of nephrologists through programs such as N-PATH [85, 86], with the aim of improving procedural outcomes [87] and raising awareness among healthcare professionals of the extent to which vascular access can alter the trajectory of chronic kidney disease.

Throughout this process, clinicians should stay attentive to the patient’s perspective, both when discussing which access is most suitable and with regard to access maintenance, which may, in some cases, be accompanied by visible changes in body appearance, leading to social stigma and arm-hiding.

## CONCLUSIONS

The nephrologist’s coordinating role in vascular access management is only achievable if adequate training in this field has been acquired. Achieving good outcomes requires repeated practice, curiosity, and creativity, particularly given that the goal is not merely to achieve a functioning access, but to consider its impact on the patient’s quality of life, in terms of both daily activities and emotional wellbeing; this is a fascinating domain precisely because it demands a personalized solution for every patient, calls for problem-solving skills, and rewards the ability to devise novel approaches. It is essential that the nephrologist takes ownership of this area, enabling continuous and meaningful exchange with the other members of the vascular access team. The results will follow, and the patient will be grateful.

## Supplementary Material

sfag366_Supplemental_Files

## Data Availability

No new data were generated or analyzed in support of this research.
