# Supplementary material for: Ten tips for the management of vascular access in hemodialysis patients
Source: Clin Kidney J. 2026 Oct 10;19(10):sfag366. doi: 10.1093/ckj/sfag366 (PMC13688669; doi:10.1093/ckj/sfag366)

TEN TIPS FOR THE MANAGEMENT OF VASCULAR ACCESS IN HEMODIALYSIS PATIENTS

Uberto Percudani[1], Marco Delsante[1], Giordano Pastori[2], Meilad Shakkour[3], Enrico Fiaccadori[1], Andrea Bandera[2]
[1] Nephrology Unit, Department of Medicine and Surgery, University Hospital of Parma, Parma, Italy
[2] Nephrology and Dialysis Unit, Hospital of Belluno, Belluno, Italy
[3] Nephrology Unit, University Hospital of Verona, Verona, Italy

TIP 1: START IN THE OUTPATIENT CLINIC

- Consider **alternative** therapeutic options: pre-emptive transplantation, peritoneal dialysis or conservative management
- Define the optimal **timing** of referral for vascular access planning, based on eGFR thresholds or the 2-year risk of kidney failure estimated with the Kidney Failure Risk Equation (KFRE)
- Recognise the central role of the **nephrologist** as leader of the multidisciplinary vascular access team

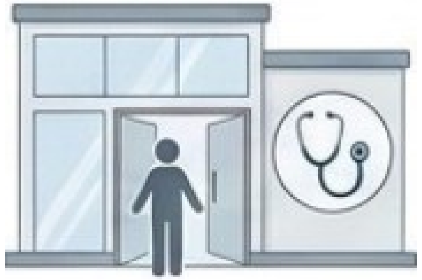

TIP 2: PLANNING MAKES THE DIFFERENCE

- "The right access, in the right patient, at the right time, for the right reasons": **patient preferences** should be central; advantages and disadvantages of each access should be discussed
- Start with a **thorough** clinical assessment including medical history, physical examination and ultrasound evaluation
- Identify at least two surgical **options** offering the lowest achievable primary failure rates

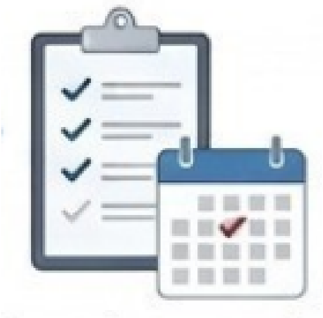

TIP 3: SAVE THE VEIN

- A simple measure that makes a real impact, particularly when patients are **educated** and engaged
- Apply it to **all patients** at risk of end-stage kidney disease, especially once the preferred site for vascular access creation has been identified
- Remember that CVCs carry a **risk** of central venous stenosis

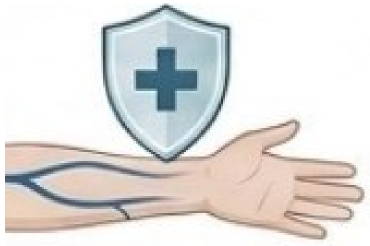

TIP 4: ALWAYS ULTRASOUND

- Rapid, non-invasive assessment of the vascular anatomy **both** at the **time** of feasibility evaluation and immediately before skin incision
- Should be performed by the **same operator** who will carry out the procedure, integrating clinical information
- **Both** the arterial and venous **compartments** should be evaluated, including vessel diameter, venous distensibility, arterial wall quality and peak systolic velocity (PSV)

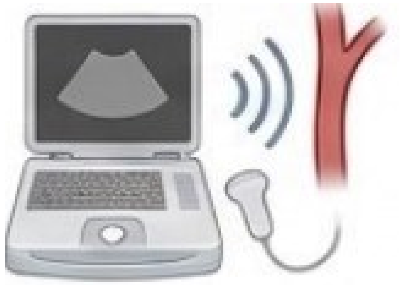

Clinical Kidney Journal

TIP 5: SURGICAL DETAILS

- Small **details** make a significant difference: longitudinal skin incision, vein-to-artery anastomotic angle, avoiding vein torsion, gentle arterial handling
- **Rescue** a failing access by introducing a dilator to relieve vasospasm and disrupt venous valves
- Note that a low flow AVF suggests an **inflow** problem whereas a pulsatile AVF suggests an **outflow** problem

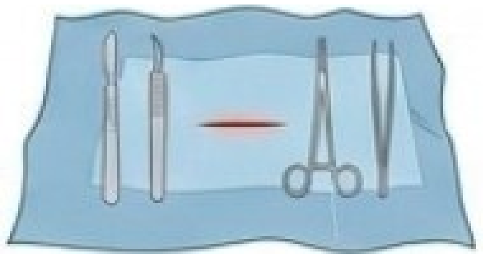

TIP 6: CARE FOR CANNULATION

- Consider different **criteria** to assess fistula maturation, «rule of 6s» does not necessarily always apply
- Cannulation should be performed by dedicated and **trained nursing** staff, skilled in fistula physical examination and ultrasound-guided puncture
- Prefer the **rope-ladder** technique to reduce complications and choose appropriate needles
- Take action to **reduce** the patient's pain and anticipatory anxiety

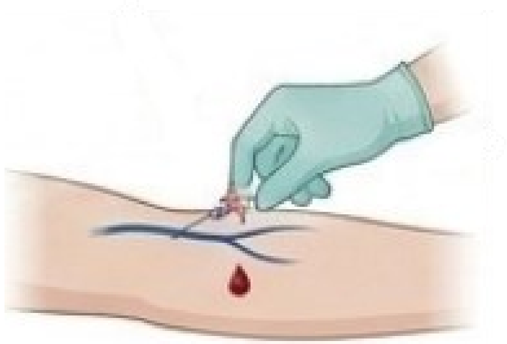

TIP 7: MONITOR TO PRESERVE

- Educate patients on the **warning** signs that warrant contacting the dialysis centre
- Nursing staff are key contributors: they should enquire about access problems, carefully **examine** the access and report any issues during dialysis
- The physician completes the monitoring process by **integrating** clinical and laboratory data to detect complications

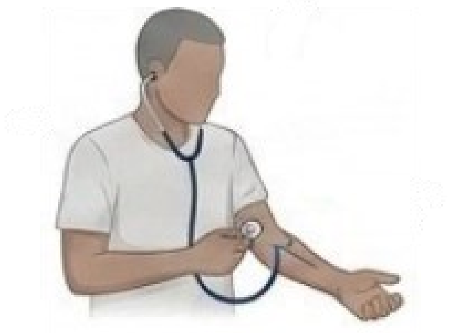

TIP 8: MANAGE COMPLICATIONS

- Recognise **urgent** conditions and treat them without delay
- Non-urgent complications allow time to decide on the best treatment strategy, especially for venous stenosis
- HAIDI and High-Flow AVF require different approaches

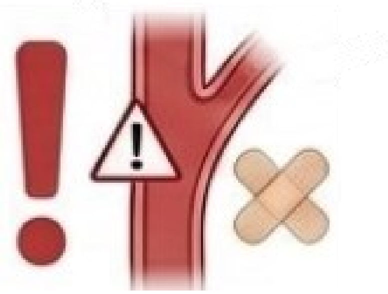

TIP 9: MAINTAIN CVCs

- **Minimise** placement, but if chosen, prefer the right internal jugular vein with correct tip position
- Frequent patient and staff CVC handling **training** to reduce infection risk
- In case of malfunction consider CT scan or **angiography** of central vessels to detect a fibrin sheath or central stenosis

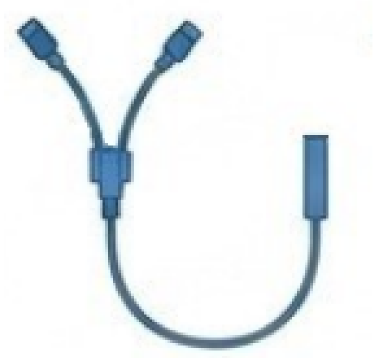

TIP 10: TEAMWORK, EDUCATION AND THE PATIENT AS A PARTNER

- Foster **collaboration** between patients, nurses, nephrologists, interventional radiologists and vascular surgeons
- Actively **participate** in discussions
- Invest in the training of **future** nephrologists

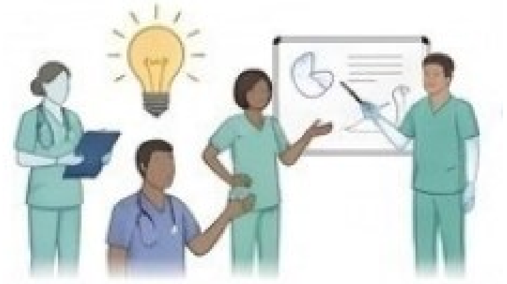

Supplement: Supplementary file 1 [file sfag366_supplemental_files.zip › 1487 ten-tips-VA management.pdf]
