# Supplementary material for: Ten tips for the management of vascular access in hemodialysis patients
Source: Clin Kidney J. 2026 Oct 10;19(10):sfag366. doi: 10.1093/ckj/sfag366 (PMC13688669; doi:10.1093/ckj/sfag366)
Supplement: Supplementary file 1 [file sfag366_supplemental_files.zip › Supplementary Table S1. Plain-language patient takeaways.pdf]

Tip	Title	Patient takeaway
**1**	Start in the outpatient clinic	Vascular access planning should begin well before dialysis is needed, so that you and your care team can choose together the best option for you. Sometimes this may even be a kidney transplant, peritoneal dialysis, or medical care focused on comfort and quality of life, without dialysis.
**2**	Planning makes the difference	There is more than one type of vascular access, and each has different pros and cons. Table 2 explains these in everyday language, so you can discuss with your doctor which option best fits your life.
**3**	Save the vein	Protecting the veins in your arms for example by avoiding needles or drips above the wrist helps keep future access options open, even before dialysis is needed. Ask your care team which arm to protect.
**4**	Always ultrasound	An ultrasound scan of your arm is a painless way to check that your veins and arteries are suitable before any access is created, helping the surgeon choose the best possible site.
**5**	Surgical details	Creating a fistula is a delicate operation. Small technical details made by an experienced surgeon can make the difference between an access that works well and one that doesn't.
**6**	Care for cannulation	Needle insertion may be uncomfortable, especially at first, but several treatments such as numbing cream, cold packs, or simple distraction techniques can help reduce pain and anxiety. It's always fine to ask for an experienced staff member if a puncture proves difficult.
**7**	Monitor to preserve	You are the first person who can notice a problem with your access. Table 3 lists warning signs such as pain, swelling, or a change in the buzzing sensation (thrill) that mean you should contact your dialysis centre.
**8**	Manage complications	If a problem with your access does occur, several treatment options exist, ranging from careful monitoring to surgery or minimally invasive procedures, depending on how urgent the issue is.
**9**	Maintain CVCs	If you have a central venous catheter, keeping it clean and dry, and letting staff care for it correctly, helps prevent infection.
**10**	Teamwork, education and the patient as a partner	Good vascular access care is a team effort involving nurses, nephrologists, surgeons, radiologists, and, most importantly, you. Your experience, including how the access affects your daily life, comfort, and body image, is a valuable part of that conversation.

Table S1: Plain-language patient takeaways for each of the ten tips discussed in the review
